# Modulation of Tumor Necrosis Factor by Microbial Pathogens

**DOI:** 10.1371/journal.ppat.0020004

**Published:** 2006-02-24

**Authors:** Masmudur M Rahman, Grant McFadden

## Abstract

In response to invasion by microbial pathogens, host defense mechanisms get activated by both the innate and adaptive arms of the immune responses. TNF (tumor necrosis factor) is a potent proinflammatory cytokine expressed by activated macrophages and lymphocytes that induces diverse cellular responses that can vary from apoptosis to the expression of genes involved in both early inflammatory and acquired immune responses. A wide spectrum of microbes has acquired elegant mechanisms to overcome or deflect the host responses mediated by TNF. For example, modulatory proteins encoded by multiple families of viruses can block TNF and TNF-mediated responses at multiple levels, such as the inhibition of the TNF ligand or its receptors, or by modulating key transduction molecules of the TNF signaling pathway. Bacteria, on the other hand, tend to modify TNF-mediated responses specifically by regulating components of the TNF signaling pathway. Investigation of these diverse strategies employed by viral and bacterial pathogens has significantly advanced our understanding of both host TNF responses and microbial pathogenesis. This review summarizes the diverse microbial strategies to regulate TNF and how such insights into TNF modulation could benefit the treatment of inflammatory or autoimmune diseases.

## Introduction

Metazoans have developed a variety of reactive mechanisms to control invading pathogens. On the other hand, microbial invaders such as viruses, bacteria, and intracellular parasites have co-evolved with their hosts to counteract the innate and adaptive responses mounted by the host. Of the many host pathways activated by pathogen invasion, pro-inflammatory cytokines play particularly significant roles in orchestrating both the early and late host responses. TNF is one such pleiotropic pro-inflammatory cytokine that plays an important role in diverse host responses such as septic shock, induction of other cytokines, cell proliferation, differentiation, necrosis, and apoptosis. TNF is expressed as either a membrane-bound or secreted ligand mainly by activated macrophages, lymphocytes, natural killer cells, and epithelial cells. Three classes of TNFs have been identified: TNFα (here called TNF), lymphotoxin-α (LT-α), and LT-β, all of which are bioactive as trimers. A TNF protein superfamily that exhibits 15%–20% identity to each other now comprises at least 20 members [1,2]. Many of the TNF-induced cellular responses are mediated by either one of the two known TNF receptors (TNFR), TNFR1 (p60), and TNFR2 (p80), both of which also belong to a larger superfamily of receptors, consisting of nearly 30 members [1,3].

The TNFR superfamily members fall into three major groups, death domain (DD)-containing receptors, decoy receptors, and TNF receptor-associated factor (TRAF) binding receptors [1]. DD-containing TNFRs (such as FAS, TNFR1, and DR3) can activate caspase cascades via DD-containing signaling intermediates, leading to apoptosis. Receptors that lack DD, such as TNFR2, contain motifs that recruit TRAF proteins. Both TNFR1 and TNFR2 and many other TNFR family members activate NF-κB (nuclear factor-κB) which is associated with cellular activation, differentiation, cytokine production, and survival signaling [1,3,4]. The TNFR superfamily members are all type I transmembrane proteins characterized by the presence of one to six hallmark cysteine-rich domains. Some members of the TNFR superfamily (FAS, TNFR1, and TNFR2) preassemble on the cell surface prior to ligand binding using the N-terminal pre-ligand binding assembly domain (PLAD) [5].

**Figure 1:**
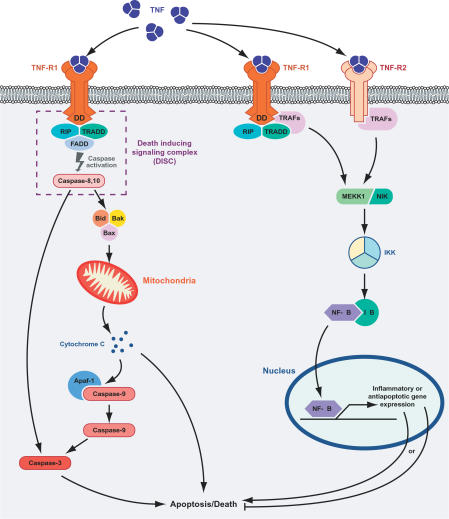
TNF-Mediated Death and Survival Pathways TNF-mediated death and survival pathways are activated following interaction with the TNFRs. The apoptotic pathway is activated through TNFR1 by forming the DISC, which activates caspase-8. Activated caspase-8 or −10 then activates the proapoptotic Bcl-2 family members, which leads to cell death by releasing cytochrome c from mitochondria and loss of MMP. The NF-κB-mediated survival pathway is activated by both TNFR1 and TNFR2. Association of TRAFs with these receptors activate signaling proteins like NIK (NF-κB inhibitor kinase) and MEKK1 (MAPK kinase 1), which activate the inhibitor of NF-κB (IkB) kinase (IKK) signalosome complex. IKK phosphorylates IkB, resulting in the degradation of the inhibitor. The free NF-κB than translocates to nucleus to induce the expression of inflammatory or antiapoptotic genes.

TNF can induce either an NF-κB-mediated survival (and proinflammatory) pathway or an apoptotic response depending on the cellular context (Figure 1). TNFR1 is thought to initiate the majority of TNF-mediated biological activities. The TNF ligand homotrimer binds to the extracellular domain of the receptor, which induces TNFR1 trimer conformational changes and the activation of the intracellular signaling pathway. TNFR1 ligand engagement leads to the release of the inhibitory protein silencer of death domains (SODD) from TNFR1 intracellular DD [6,7]. Release of SODD allows binding of TRADD (TNFR1-associated death domain protein) to the DD and recruits additional adapter proteins such as RIP1 (receptor interacting protein), TRAF2, and cIAP1 (cellular inhibitor of apoptosis) to form complex I. Complex I transduces signals leading to NF-κB translocation to the nucleus. Later, RIP1, TRADD, and TRAF2 dissociate from TNFR1 and recruit FADD (FAS-associated death domain protein) and caspase 8 to form complex II. In the absence of NF-κB activity from complex I, complex II can initiate caspase-8 activation, which leads to cell death [8,9]. On the other hand, NF-κB inhibits cell death through upregulation of antiapoptotic genes such as cellular FLICE-like inhibitory protein (c-FLIP), cIAP1, cIAP2, TRAF1, and TRAF2, which are recruited to complex II and inhibit caspase activation [10].

TNFR2 does not contain a cytoplasmic death domain and cannot directly engage the apoptotic machinery, and thus its precise involvement in TNF-mediated cell death is controversial. It can enhance the cell death signal of TNFR1, possibly through TRAF2 degradation and enhanced recruitment of FADD and RIP to TNFR1 [11]. TNFR2 also plays an important role in antiviral response by inducing cellular necrosis [12,13]. TNFR2 itself can induce TNF-dependent apoptosis and cell death, as demonstrated using cytotoxic T lymphocytes from TNFR1 knockout animals, possibly by recruitment of FADD to TNFR2 via RIP1 and TRAF2 together with some still-unidentified adapter molecules [14].

The TNF-induced NF-κB–mediated survival pathway can be activated independently by either TNFR1 or TNFR2 [15]. In response to TNF, complex I signals through some other scaffolding and signaling proteins, such as NIK (NF-κB inducing kinase) and MEKK1 (MAPK kinase-1), which converge on the IκB kinase (IKK) signalosome complex and activate NF-κB. Depending on stimuli such as viral or bacterial infection, exposure to proinflammatory cytokines, mitogens, growth factors, and stress inducing agents, IKK-α/IKK-β can phosphorylate IκB, resulting in proteolytic degradation of the inhibitor and translocation of NF-κB to the nucleus [16]. NF-κB enhances cell survival by upregulating expression of antiapoptotic genes such as members of the Bcl2 family (Bcl-xL and A1/Bfl-1), cellular inhibitors of apoptosis (c-IAP1, c-IAP2, and XIAP), TRAF1 and TRAF2, and the FLICE-inhibitory protein cFLIP. In some cases, activation of NF-κB is also associated with induction of apoptosis by enhancing expression of proapoptotic cytokines [17,18]

## Inhibition and Modulation of TNF by Viruses

**Figure 2:**
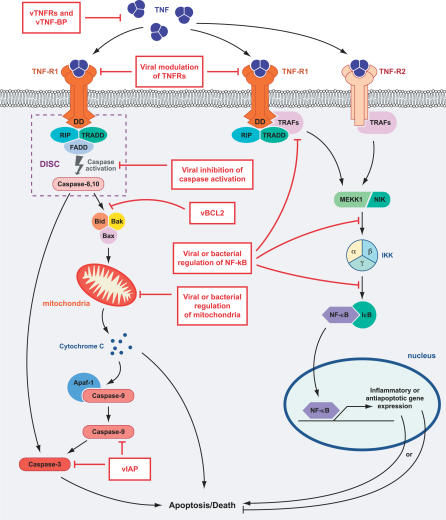
Different Strategies for Inhibiting TNF by Pathogens Pathogens have evolved diverse strategies to target almost every step of TNF biology. Virus-encoded proteins inhibit TNF-mediated responses by directly binding to TNF with secreted soluble decoy TNFR (vTNFRs) and vTNFBPs, downregulating the cellular death receptors, interacting with the TNFR-associated factors, blocking caspase activation, and regulating the apoptotic checkpoint function of mitochondria. Viruses also regulate the pathways leading to TNF-mediated activation of NF-κB. Bacteria and other pathogens can express proteins that regulate TNF-mediated responses by activating or inhibiting NF-κB at different levels of signaling that range from the death receptor to nuclear localization of NF-κB.

TNF orchestrates powerful anti-microbial responses by a variety of mechanisms, including the direct killing of infected cells (cytolysis), induction of apoptosis, inhibition of intracellular pathogen replication, and upregulation of other diverse host responses. Using TNF or TNFRs-deficient mice, it has been demonstrated that they are essential for survival of infections with bacterial pathogens such as *Listeria monocytogens, Mycobacterium tuberculosis, M. avium, Salmonella typhimurium,* intracellular parasites such as Leishmania major or *Trypanosoma cruzi,* and viruses such as herpes simplex virus (HSV-1), mouse cytomegalovirus (MCMV), or lymphocytic choriomeningitis virus (LCMV)[2,4,13]. TNF and TNFR signaling pathway is required for differentiation of T cells, induction of cytokines and chemokines, recruitment of leukocytes, and development of granulomas that are capable of controlling virulent bacterial infection. Deficiency of TNF or TNFR network delays granuloma formation and affects several other components of the innate and adaptive immune system including activation of dendritic cells, natural killer cells, and differentiation of T and B cells. Thus, TNF and TNFR network provided powerful selection pressure for viruses and other pathogens to evolve strategies to combat the TNF-mediated responses to infection. As illustrated in Figure 2, many viruses have acquired strategies to neutralize TNF by targeting almost every step of TNF biology, ranging from direct binding and inhibition of the ligand or receptor, to modulation of various downstream signaling events [19,20]. Table 1 represents a spectrum of the viral factors that inhibit TNF or modulate TNF signaling.

**Table 1:**
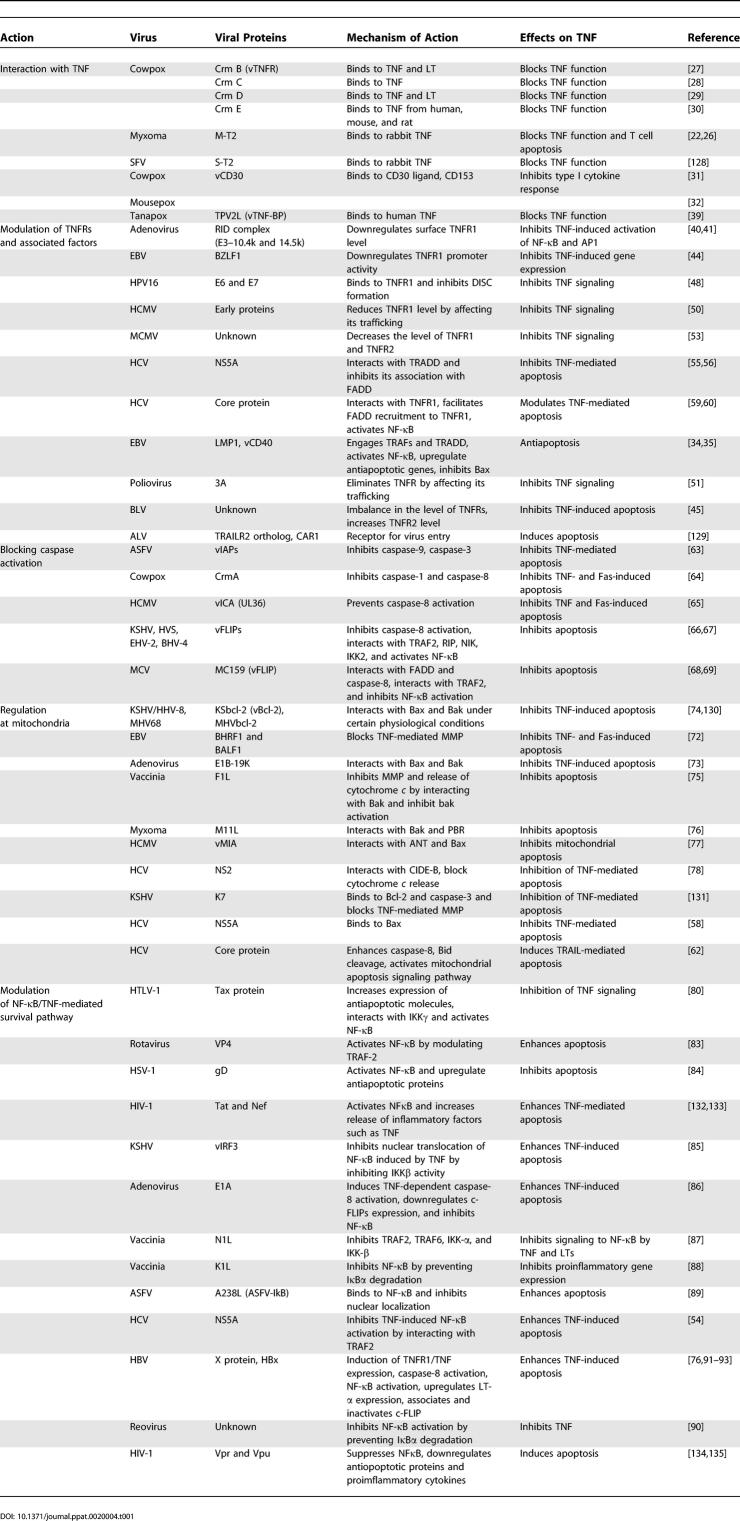
Viral Inhibition and Modulation of TNF

## Viral TNFR Homologues

The first-identified TNF inhibition strategy deployed by viruses was revealed by the discovery of encoded soluble TNFRs homologs which function by binding and sequestering extracellular TNF. Among these vTNFRs are the T2-like family members encoded by Leporipoxviruses and the cytokine response modifier (Crm) family members encoded by Orthopoxviruses [21,22]. The myxoma-virus-encoded M-T2 protein is a glycosylated, dimeric, secreted protein that specifically inhibits rabbit TNF [23,24]. The pathogenicity of myxoma virus in domestic rabbits is attenuated when the M-T2 gene is deleted from the viral genome [25]. The intracellular form of M-T2 protein is also able to inhibit apoptosis in virus-infected lymphocytes. The first two N-terminal cysteine-rich domains are responsible for anti-apoptotic properties, while at minimum the first three cysteine-rich domains are required to inhibit TNF [26]. Related TNFR orthlogs, designated CrmB, CrmC, CrmD, and CrmE, have been characterized from members of the orthopoxvirus genus [27–30]. Another poxvirus encoded ortholog of TNFR family members, vCD30, has been identified in cowpox and mousepox viruses [31,32]. vCD30 binds to CD30L/CD153 with high affinity, inhibits the ability of CD30L to signal via cell surface CD30, and also inhibits type I cytokine responses in a murine model of antigen-induced granuloma [32].

Epstein-Barr virus (EBV) latent infection membrane protein 1 (LMP1) mimics a constitutively activated TNFR member, CD40. LMP1 is essential for EBV conversion of infected B lymphocytes into perpetually proliferating lymphoblasts and is expressed in EBV-associated lymphoproliferative disease, Hodgkin disease, and nasopharyngeal carcinoma [33]. Two motifs of LMP1 located in the carboxy-terminal cytoplasmic domain, designated CTAR1 (C-terminal activation domain) and CTAR2, activate both canonical (IKKβ-dependent) and noncanonical (NIK/IKKα-dependent) NF-κB pathways by engaging different TRAFs. [34]. This activation mediates the antiapoptotic activity of LMP-1 by upregulation of the expression of antiapoptotic factors such as Bcl-2, Bfl-1, and A20, which are potent inhibitors of Bax [35].

Some viruses exploit TNFR family members as receptors to enter into cells. HSV-1 glycoprotein D (gD) interacts with HVEM (herpes virus entry mediator) to enter into resting T cells, monocytes, and immature dendritic cells [36]. A TNFR ortholog (UL 144 orf) encoded by human CMV, which is closest in sequence to the extracellular domain of human HVEM and TRAILR2, binds BTLA (B and T lymphocyte attenuator) and inhibits T cell proliferation by mimicking the inhibitory cosignaling function of HVEM [37]. Recently, another TNFR family member, designated equine lentivirus receptor-1, has been identified as cellular receptor for the entry of equine infectious anemia virus, a member of lentiviruses family, into monocytes and macrophages [38].

## Viral TNF-Binding Proteins

A new class of TNF binding protein has been recently identified from Tanapox virus, a member of the Yatapoxvirus genus of poxviruses [39]. This vTNF-BP is encoded by the TPV gene 2L, and related orthologs are present in other members of the Yatapoxvirus (YLDV and YMTV), swinepox, and deerpoxvirus. The 2L protein exhibits some sequence identity (25%) to the α1, α2, and α3 domains of the cellular MHC class I molecules, but, unlike the cellular counterpart, lacks a transmembrane domain. TPV-2L binds to human TNF with very high affinity (Kd = 43 pM), but this protein failed to interact with any other human cytokine or TNF from other species. The discovery of this novel group of vTNF-BPs suggests that there could still be unidentified classes of cellular TNF-BPs, too.

## Modulation of TNF Receptors and Associated Factors by Viruses

The adenovirus early transcription region 3 (E3) encodes at least seven proteins, five of which block the acquired or innate immune response. Three of these, Ad E3–14.7K, Ad E3–10.4K, and Ad E3–14.5K, impose inhibitory effects on the TNF pathway [40]. Two of these proteins, 10.4K (RIDα) and 14.5K (RIDβ), form a heterotrimeric complex in the plasma membrane known as RID (receptor internalization and degradation), which inhibits signaling through TNFR1 [40]. RID downregulates surface TNFR1 levels by reducing the assembly of TNFR1 signaling complex and thus inhibiting TNF induced activation of NF-κB. In terms of the NF-κB pathway, RID blocks the association of members of the IKK complex, as well as the protein kinase RIP, with the TNFR1 [40]. In a recent study it has been demonstrated that RIDβ directly interacts with TNFR1, and its tyrosine sorting motif plays a major role in downregulation of TNFR1 by a clathrin-dependent process involving μ2 and dynamin, followed by degradation of TNFR1 via an endosomal/lysosomal pathway [41]. In addition to TNFR1, RID can degrade other death receptors such as Fas, and in conjunction with E3–6.7K protein it can also degrade TRAIL receptor 2 [42,43].

The EBV-encoded immediate-early gene product BZLF1 (also called Zta, ZEBRA, or EB1) prevents cellular responses to TNF, including TNF-induced cell death [44]. During reactivation of the EBV lytic cycle, BZLF1 reduces TNF-R1 promoter activity and thus downregulates TNF-R1 protein expression levels. Mutational analysis of BZLF1 revealed that inhibition of TNF-R1 promoter activity requires both the transactivation and the DNA binding domains of BZLF1, suggesting that BZLF1 may bind to and activate the promoter of a gene that encodes a repressor of the TNF-R1 promoter [44]. Bovine leukemia virus (BLV), a type C retrovirus, induces TNFR2, but not TNFR1, by a yet unknown mechanism in PBMC from BLV-infected cattle, which results in resistance to TNF-induced apoptosis, possibly by activating antiapoptotic genes in an NF-κB–dependent fashion [45].

Small DNA viruses, such as human papillomaviruses (HPVs) are the major cause of cervical cancer (>90%) and a significant number of head and neck cancers [46]. They infect various human epithelial tissues, and have acquired mechanisms to inhibit TNF-induced apoptosis. HPV16 encoded two oncogene products, E6 and E7, which can stimulate cell cycle progression by binding to the tumor suppressor proteins or negative regulator of the cell cycle p53 and retinoblastoma (Rb) protein, respectively [47]. Inactivation of these proteins leads to deregulated entry of cells into S phase and maintenance of a favourable environment for viral DNA replication. Both E6 and E7 can also associate with other proteins involved in cell proliferation and apoptosis. HPV16 E6 protein binds to TNFR1 and affects the transmission of pro-apoptotic signals triggered by TNF [48]. E6 binds to the C-terminal 41 amino acids of TNFR1 and inhibits binding of TRADD to TNFR1 and thereby blocks formation of the death-inducing signaling complex (DISC). This inhibition subsequently blocks transmission of apoptotic signals by inhibiting the activation of initiator caspases such as caspase 8. E6-mediated protection against TNF-induced apoptosis occurs in cells of different species (mouse and human) and tissues (fibroblast, osteosarcoma, and histiocyte/monocyte). Both E6 and E7 of HPV16 increased the transcription of cIAP1 and cIAP2 by upregulation of NF-κB–expression and confer resistance to TNF in human keratinocytes [49].

Human and murine CMV have also developed mechanisms to evade a TNF-induced antiviral state by dysregulating TNFRs. HCMV infection of THP1 cells reduced the level of TNFR1 on the cell surface by accumulating the receptor pool in the trans-Golgi network [50]. Time course analysis and drug inhibition studies suggest that viral early gene products may target trafficking of TNFR1 [50]. Poliovirus noncapsid protein 3A also affects the intracellular trafficking of TNFR and induces TNF resistance by eliminating TNFRs from the plasma membrane [51]. However, 3A-protein–mediated inhibition of ER to Golgi traffic of TNFR was limited to poliovirus and coxsackievirus B3 [51,52]. MCMV infection of bone marrow-derived macrophages inhibited TNF-induced ICAM-1 surface expression and mRNA expression in infected cells via expression of immediate early and/or early viral genes [53]. MCMV infection blocked TNF-induced nuclear translocation of NF-κB, which decreased the level of both TNFR1 and TNFR2.

The hepatitis C virus (HCV) nonstructural protein 5A (NS5A) is a multifunctional phosphoprotein that utilizes multiple mechanisms to inhibit both extrinsic and intrinsic apoptotic stimuli [54]. Using NS5A transgenic mice, it has been demonstrated that NS5A interacts with TRADD, inhibiting its association with FADD and TNF-mediated apoptosis, resulting in persistent infection [55,56]. NS5A also binds to the TNFR1 signaling complex through its interaction with TRAF2, and subsequently inhibits TRAF2-dependent NF-κB activation, thereby sensitizing the cells to TNF-induced cytotoxicity. However, the sensitivity of cells expressing NS5A to TNF was not affected [57]. The inhibition of intrinsic apoptotic signals is mediated by the putative BH (Bcl-2 homology) domain of NS5A, which allows it to bind to the pro-apoptotic protein Bax, rendering cells refractile to certain pro-apoptotic agonists [58]. HCV core protein has been reported as both inducer and inhibitor of TNF-mediated apoptosis. In some human and mouse cell lines, HCV core protein interacts with TNFR1 or LTβR and activates the NF-κB pathway [59]. Core protein can also facilitate FADD recruitment to TNFR1 and sensitize cells to TNF-induced apoptosis [60]. However, HCV core-protein–mediated suppression of TNF-induced apoptosis has also been reported [61]. One recent study demonstrated that HCV core protein also induces TRAIL-mediated apoptosis in Huh7 cells through sequential induction of DISC formation [62].

## Viral Inhibition of TNF-Induced Apoptosis

Activation of caspases and release of cytochrome c from the mitochondria generally lead to the induction of apoptosis and cell death. A number of viral proteins have been shown to inhibit caspases or intervening at the mitochondrial checkpoint to prevent TNF-mediated apoptosis. Proteins that inhibit caspase activation include vIAP from ASFV, CrmA from poxvirus, vICA from HCMV, vFLIPs from several γ-herpesviruses, and its ortholog MC159 from MCV [63–69]. One group of antiapoptotic proteins known as vBcl-2 from γ-herpesviruses, adenoviruses, and poxviruses inhibit proapoptotic Bax and Bak, and block mitochondrial apoptosis. Some viruses that lack vBcl-2 instead encode mitochondria-localized protein such as F1L from vaccinia, M11L from myxoma, and vMIA from HCMV, which inhibit apoptosis by preventing depolarization of the mitochondrial membrane potential (MMP) and stopping the release of cytochrome c [70–78].

## Modulation of NF-κB by Viruses

NF-κB is a critical regulator of the immediate early pathogen response, playing an important role in promoting inflammation, and in the regulation of cell proliferation, activation and survival [79]. NF-κB thus provides an attractive target to various microbial pathogens for modulating host TNF-mediated events.

The Tax transactivator oncoprotein of human T cell leukemia virus type I (HTLV-I) persistently activates NF-κB signaling pathways, resulting in the deregulation of cellular gene expression and immortalization of HTLV-I-infected T cells [80]. Tax interacts with IKK-γ and stimulates the catalytic activity of IKK-α and IKK-β, which degrades IκB and enhances the activation of NF-κB [81]. Tax also interacts with and blocks tristetraprolin repressor, an inhibitor of TNF expression, and indirectly increases TNF expression in macrophages [82]. Rotavirus capsid protein VP4 contains a conserved TRAF-binding motif, and is responsible for NF-κB activation and the inhibition of TNF-mediated death signaling by engaging the TRAF2-NIK signaling pathway [83]. HSV-1 encoded gD inhibits TNF-mediated apoptosis in the U937 monocytoid cells by activation of NFκB and upregulation of some of the downstream antiapoptotic proteins, such as FLIP and cIAP2 [84].

Viruses also can inhibit NF-κB activation by different methods, which can result in increased sensitivity to TNF-induced apoptosis. KSHV-encoded viral interferon regulatory factor 3 (vIRF3) inhibits the activation of NF-κB induced by TNF [85]. In 293T cells, vIRF3 inhibits IKKβ activity, resulting in reduced IκB phosphorylation and inhibition of NF-κB activity and thus sensitizes cells to TNF-induced apoptosis [85]. Adenovirus E1A protein also sensitizes cells to TNF-mediated apoptosis. E1A inhibits c-FLIPs expression, which results in TNF-dependent caspase-8 activation in the DISC [86]. Vaccinia virus encoded protein N1L, a viral virulence factor, inhibits signaling to NF-κB via both TNF and LT. This N1L-mediated inhibition of NF-κB occurs by association with IKK-γ and inhibition of IKK-α and IKK-β [87]. N1L also inhibits IRF3 signaling and thus might play a broad role as viral immunomodulator of innate immunity. Another vaccinia encoded protein, K1L, inhibits NF-κB activation by preventing IκBα degradation, probably by interfering directly with IKK to prevent phosphorylation or indirectly by hampering kinases that act upstream of IKK [88]. ASFV A238L, which is an ankyrin-repeat–containing homolog of host IκB (ASFV-IκB), binds to NF-κB following degradation of host IκB and inhibits the nuclear transportation of NF-κB [89]. In contrast, Reovirus induces apoptosis by regulating NF-κB at two distinct levels. In human epithelial HEK293 cells, reovirus activates NF-κB to induce apoptosis early after infection. At later times, the reovirus inhibits NF-κB activation by preventing degradation of IκBα and inhibiting TNF-mediated apoptosis [90].

Induction of apoptosis in HBV-infected hepatocytes is mediated by the viral X protein (HBx). HBx-dependent activation of p38MAP kinase and JNK pathways leads to the activation of both TNFR1/TNF and Fas/FasL [91]. HBx also binds and inactivates c-FLIP and activates NF-κB through induction of LTα and TNF, both of which induce apoptosis [92,93].

## Modulation of TNF by Bacteria and Parasites

Bacterial mechanisms of inhibiting TNF-mediated responses differ significantly from that of viruses. To date, no bacterial or parasite protein has been reported that directly binds and inhibits TNF, TNFR, or their associated factors. Instead, they rely on indirect mechanisms to modulate TNF signaling, for example by reducing the synthesis of inflammatory mediators, cytokines, and the modulation of signaling pathways such as NF-κB or MAPK (Figure 2). Table 2 lists some of the known TNF modulating factors produced by bacteria and other nonviral pathogens.

**Table 2:**
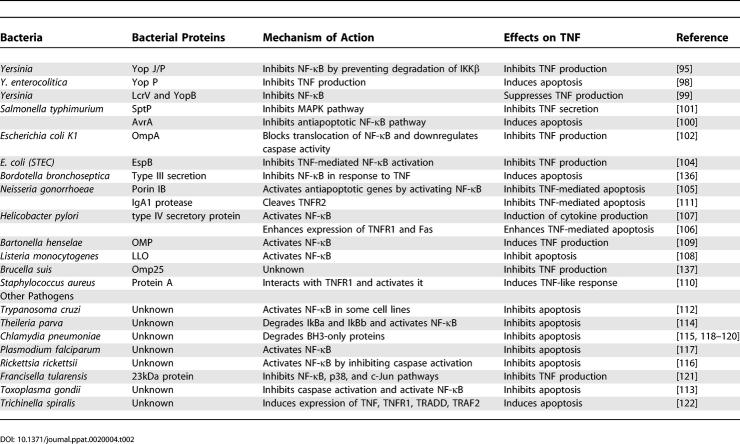
Modulation of TNF by Bacteria and Parasites

Bacterial pathogens generally mediate their interactions with host cells via preformed soluble small-molecule or peptide effectors secreted by type III secretion system, a multicomponent translocation apparatus that spans the bacterial cell wall from the interior of the bacterium into the external environment [94]. The best-studied effector proteins are from *Yersinia,* and known as Yops (Yersinia outer proteins). Among the six Yop effector proteins, YopP/YopJ is involved in the inhibition of NF-κB and MAPK pathways, which results in the downregulation of cytokines such as TNF, chemokines, and adhesion molecules. This then inhibits the recruitment and activation of macrophages and natural killer cells to the site of infection [95]. YopJ is a cysteine protease (structurally related to the ubiquitin-like protease family of proteins) that can remove K63- and K48-linked polyubiquitin chains and inhibit proteasomal degradation of 1κBα, resulting in inhibition of NFκB signaling. YopJ also removes ubiquitin moieties from TRAF2 and TRAF6 [96,97]. Recently it has been demonstrated that YopP from Yersinia enterocolitica also induces apoptosis in murine dendritic cells and inhibits TNF production [98]. Two other type III secretory proteins from *Yersinia pestis,* Low calcium response V (LcrV) or V antigen and YopB inhibit production of TNF from murine peritoneal macrophages by inhibiting the transcription factor NF-κB after LPS treatment [99].

An ortholog of YopJ, AvrA from *Salmonella typhimurium,* also demonstrates potent inhibitory action towards the NF-κB pathway but does not seem to affect MAPK activation [100]. Unlike YopJ, AvrA in HeLa epithelial cells potently inhibits TNF-induced activation of the NF-κB pathway by inhibiting translocation of the p65 subunit of NF-κB. Another type III secretion protein, SptP from *S. typhimurium,* is also involved in host modulation involving the MAPK pathway by inhibiting Raf activation, which ultimately attenuates the secretion of TNF from infection-activated macrophages [101].

Escherichia coli K1 outer membrane protein A (OmpA) in infected monocytes suppresses the production of cytokines such as TNF by inhibiting IκB phosphorylation and blocking the translocation of NF-κB to the nucleus [102]. OmpA also induces expression of antiapoptotic protein Bcl-xL to promote the survival of monocytes and macrophages [103]. Suppression of NF-κB activation and downregulation of genes involved in inflammatory and immune responses is the most common mechanism used by other pathogenic E. coli strains. The Shiga toxin-producing E. coli (STEC), enterohemorrhagic *E. coli,* and enteropathogenic E. coli all interfere with NF-κB–activation initiated by TNF. EspB (*E. coli–*secreted protein B), a component of the type III secretion system, is also involved in the inhibition of NF-κB activation and proinflammatory cytokine production [104].

Some bacterial proteins activate NF-κB, which inhibits TNF-mediated apoptosis by upregulation of antiapoptotic genes, or induces TNF production to enhance apoptosis. The outer membrane protein, porin, from *Neisseria gonorrhoeae,* increases the transcription of several host antiapoptotic genes, including *bfl-1, cox-2,* and *c-IAP-2,* by the activation of NF-κB, and thus protects human urethral epithelial cells from apoptosis [105]. Infection with *Helicobacter pylori,* the main causative agent of chronic active type B gastritis, enhances the expression of TNFR1 and Fas and induces apoptosis of gastric epithelial cells [106]. Proteins encoded by the cag pathogenicity island of H. pylori are required for NF-κB activation, which enhances the production of TNF and other pro-apoptotic cytokines [107]. Listeria monocytogenes virulence protein Listeriolysin O (LLO) and Outer membrane protein (OMP) from Bartonella henselae both activate NF-κB and induce antiapoptotic signaling, which prolongs bacterial survival [108,109].

Bacteria can also use host TNFRs to mediate pathogenicity. A recent study has demonstrated that TNFR1 is a receptor for protein A from *Staphylococcus aureus,* a pathogen associated with pneumonia and sepsis. Activation of TNFR1 by protein A induces TNF-like responses which are associated with the pathogenesis of staphylococcal pneumonia [110]. Extracellular IgA1 protease from Neisseria gonorrhoeae is capable of inhibiting the TNF-mediated apoptosis of the human myelo-monocytic cell line U937 [111]. This proteolytic enzyme cleaves TNFRII but not TNFRI. Since TNFRII also can activate NFκB and induces apoptosis, inactivation of TNFRII could lead to direct inhibition of apoptosis.

Like virus and bacteria, parasites can also modulate NF-κB function and regulate host immune responses. They can either activate NF-κB by degrading the IκB or inhibit NF-κB activation by blocking the degradation of IκB. Protozoan parasites such as *Trypanosoma cruzi, Toxoplasma gondii, Theileria parva,* and other pathogens such as *Chlamydia pneumoniae, Plasmodium falciparum,* and Rickettsia rickettsii all activate NF-κB and inhibit apoptosis to enhance parasite replication [112–117]. Recent studies demonstrated that Chlaydia infection results in the degradation of BH3-only proteins such as Bim, Puma, Bad, Bik, Bmf, Noxa, and tBid, which inhibit proapoptotic Bax and Bak [118–120]. Degradation of these proapoptotic factors leads to protection of infected cells against apoptotic stimuli such as TNF [120]. A 23 kDa protein, which is upregulated during intracellular infection from the intracellular pathogen *Francisella tularensis,* inhibits TNF secretion from the murine macrophage like cell line J774A.1 by blocking the degradation of IκB and inhibiting NF-κB [121]. It has been demonstrated that the intracellular parasite nematode Trichinella spiralis can induce expression of TNF, TNFR1, TRADD, caspase-3, caspase-8, TRAF-2, and RIP in infected muscle cells, resulting in induction of either apoptosis or the transformation of muscle cells to nurse cells [122].

## Anti-TNF Therapy: Clues from Pathogens?

Although TNF plays a major role in growth regulation, cell differentiation, and response to microbial infections, its inappropriate overexpression has been implicated in the pathogenesis of a wide spectrum of human disorders, such as autoimmunity (e.g., multiple sclerosis, rheumatoid arthritis, inflammatory bowel disease), allergy, septic shock, allograft rejection, and insulin resistance. TNF derived from mast cells also plays a crucial role in initiation of inflammation, particularly in the case of rheumatoid arthritis [123]. TNF may also exert tumor-promoting activity [124]. A recent study has demonstrated that the PLAD domain of TNFR1 is critical in TNF response, because mutations in PLAD reduce NF-κB activation and cause TNFR-associated periodic syndrome, an autoinflammatory syndrome [125]. Protein therapeutics containing only the PLAD domain can effectively prevent TNFR signaling and potently inhibit arthritis [126].

Many approaches have been investigated to inhibit TNF activity for the treatment of various inflammatory/autoimmune diseases (e.g., rheumatoid arthritis, Crohn disease, and inflammatory bowel disease). The currently commercially available TNF antagonists are infliximab (a chimeric mouse/human monoclonal anti-TNF antibody), etanercept (a soluble fusion protein combining two p75 TNFRs with an Fc fragment of human IgG1), and adalimumab (a humanized monoclonal anti-TNF antibody). Although they have shown to be partially effective in clinical trails, still more needs to be learned in terms of the biology of TNF. These current inhibitors need to be delivered at high doses, and some adverse events have been reported, so that the long-term safety of all these molecules is not thoroughly understood [127]. The investigation of TNF inhibition mechanisms by pathogens may provide novel therapeutic insights. In particular, TNF inhibitors derived from viral pathogens, which operate at relatively low concentration within the infected host, offer new therapeutic strategies for reducing the pathologic consequences of excessive TNF expression in inflammatory disorders.

## Abbreviations

BLV: bovine leukemia virus
c-FLIP: cellular FLICE-like inhibitory protein
cIAP1: cellular inhibitor of apoptosis
CMV: cytomegalovirus
Crm: cytokine response modifier
DD: death-domain
DISC: death-inducing signaling complex
E3: early transcription region 3
EBV: Epstein-Barr virus
EspB: (*E. coli*–secreted protein B)
FADD: FAS-associated death domain protein
HCV: hepatitis C virus
HPV: human papillomavirus
HSV-1: herpes simplex virus 1
HTLV-I: human T cell leukemia virus type I
HVEM: herpes virus entry mediator
LcrV: Low calcium response
LLO: Listeriolysin O
LMP1: latent infection membrane protein 1
MMP: mitochondrial membrane potential
OMP: outer membrane protein
OmpA: outer membrane protein A
PLAD: pre-ligand binding assembly domain
RID: receptor internalization and degradation
RIP: receptor interacting protein
SODD: silencer of death domains
TNF: tumor necrosis factor
TNFR: TNF receptors
TRADD: (TNFR1-associated death domain protein
TRAF: TNF receptor-associated factor
vIRF3: viral interferon regulatory factor 3
Yops: Yersinia outer proteins
